# You only look once-based neural network and grading method for peach leaf shot hole disease detection under natural conditions

**DOI:** 10.3389/fpls.2026.1808724

**Published:** 2026-07-16

**Authors:** Bohao Liu, Xiu Wang, Jianjun Hao, Wei Zou, Hongwei Yan, Cuiling Li, Changyuan Zhai

**Affiliations:** 1 Intelligent Equipment Research Center, Beijing Academy of Agriculture and Forestry Sciences, Beiing, China; 2 College of Mechanical and Electrical Engineering, Hebei Agricultural University, Baoding, China; 3 Beijing PAIDE Science and Technology Development Co., Ltd., Beijing, China; 4 National Engineering Research Center of Intelligent Equipment for Agriculture (NERCIEA), Beijing, China

**Keywords:** deep learning, disease detection, natural environment, peach leaf shot hole disease, YOLO

## Abstract

The frequent occurrence of peach leaf shot hole disease severely affects peach yield and fruit quality. Under natural conditions, the detection accuracy of existing methods is often compromised by substantial variability in disease characteristics, complex environmental backgrounds, and dense leaf coverage that obscures lesion features. To improve detection performance under such conditions and to enable quantitative assessment of disease severity, this study proposes a YOLO–PSH (You Only Look Once–peach shot hole) model for peach leaf shot hole disease detection. The proposed model addresses three critical challenges: multi-scale feature extraction, suppression of complex background interference, and robustness to overlapping and occluded leaves. First, the GhostConv and AConv modules are introduced to reduce computational redundancy and enhance feature compression while preserving fine-grained details. Second, the A2C2f and RepNCSPELAN4 modules, combined with a regional attention mechanism and parameter reorganization strategy, are used to strengthen semantic representation in lesion regions and mitigate background interference. Third, a multi-scale feature fusion neck that integrates SPPELAN (spatial pyramid pooling with efficient layer aggregation network) and C3k2 is designed to improve adaptability to overlapping and occluded disease features. In addition, a disease severity grading method is developed based on detection results to enable regional severity assessment. To evaluate model performance, a PSHData (peach shot hole data) dataset was constructed, consisting of 800 images of peach leaves affected by shot hole disease and containing 9, 183 annotated instances. Experimental results demonstrate that the proposed YOLO–PSH model achieves an average detection accuracy of 81.49%. The model contains 1.82 million parameters, requires 8.71 GFLOPs, and has a model size of 7.00 MB. The average inference time is 7.1 ms per image. For disease severity grading, the classification accuracies for healthy, mild, moderate, and severe categories are 81.82%, 60.00%, 72.22%, and 91.67%, respectively. These results indicate that YOLO–PSH exhibits strong robustness and adaptability under natural conditions, demonstrating its potential for accurate detection and severity assessment of peach leaf shot hole disease across varying scales, complex backgrounds, and dense leaf occlusion scenarios.

## 1 Introduction

Peach trees are among the world’s most economically important fruit crops, valued for their desirable flavor and high nutritional content. China has a peach cultivation area of approximately 890, 000 hectares and an annual production of approximately 15.993 million tons, ranking first globally in both planting area and yield (Wang, 2021), with a total industrial output value approaching 100 billion yuan (Wang et al., 2026). However, during cultivation and management, peach trees are affected by environmental factors such as climate, temperature, humidity, and growing conditions, making peach leaves susceptible to infection by shot hole disease pathogens. This disease reduces fruit yield and quality and causes substantial economic losses.

Conventional identification of peach leaf shot hole disease relies primarily on manual assessment by growers, which is subjective, time-consuming, labor-intensive, and prone to misjudgment (Xu and Chen, 2023). Inexperienced growers, in particular, may inaccurately assess disease severity, leading to the overuse or misuse of pesticides. This not only reduces the effectiveness of disease control but can also exacerbate disease spread while causing environmental pollution and additional economic losses (Li et al., 2021). Accurate and efficient identification of peach leaf shot hole disease, reliable severity assessment, and precision pesticide application based on disease severity are critical for effective orchard management. Therefore, the development of precise and lightweight detection and grading methods for peach leaf shot hole disease under natural conditions is of significant practical importance.

With the continuous advancement of science and technology, visual image-based disease detection methods have been extensively studied and are gradually replacing manual inspection. Early approaches to fruit tree leaf disease detection primarily relied on machine learning techniques, in which recognition models were constructed using handcrafted features such as color, texture, and shape extracted from disease images (Guo et al., 2024). For example, Bai et al. proposed an improved fuzzy C-means algorithm for segmenting cucumber septoria leaf spot, achieving an average missegmentation rate of 0.12%; however, the method is prone to local missegmentation and does not address disease severity classification (Bai et al., 2017). Barbedo et al. developed a semi-automatic image segmentation algorithm for plant leaf diseases that distinguishes diseased from healthy regions, but it operates only under ideal conditions and does not support fully automatic detection (Barbedo, 2016). Sannakki et al. proposed a disease identification and grading method based on image processing and fuzzy logic for pomegranate leaf diseases, enabling both disease recognition and severity classification (Sannakki et al., 2013). Zhang et al. constructed an apple disease identification model using a region-growing algorithm and a support vector machine classifier, achieving a maximum recognition accuracy of 94.78% (Zhang et al., 2017). Despite their effectiveness, these conventional machine learning methods rely heavily on manual feature extraction and prior knowledge, limiting their ability to capture complex image characteristics. Their performance degrades significantly under natural conditions with complex backgrounds.

With the rapid development of deep learning, these techniques have been increasingly applied to fruit tree disease detection owing to their high accuracy and efficiency. In peach disease detection, Yan et al. proposed an improved Xception-based classification method for identifying peach diseases under natural conditions by incorporating L2 norm and mean regularization terms, achieving an accuracy of 93.85% (Yao et al., 2021). However, this approach does not address disease localization, complex backgrounds, or occlusion. Wang et al. proposed an improved You Only Look Once version 5 (YOLOv5) network to reduce missed detections and false positives in peach leaf disease detection, achieving an average detection precision of 94.8% (Wang and Hu, 2025); however, the method is primarily suitable for natural environments with simple backgrounds and minimal leaf occlusion. Yao et al. combined mask region-based convolutional neural network (Mask R-CNN) with the focal loss function to accurately identify and segment peach branch disease regions under natural conditions with a single background, effectively addressing data imbalance (Yao et al., 2022). However, their dataset consisted mainly of close-up images of local lesions, limiting the model’s adaptability to multi-scale disease detection in complex orchard environments. Li et al. proposed an improved YOLOv8 nano (YOLOv8n)-based detection algorithm to reduce the missed detection rate of small disease targets using a self-constructed dataset under ideal conditions, achieving an accuracy of 80.8% (Li et al., 2025); however, the method does not consider disease localization under occlusion or disease severity grading. Ji et al. proposed a lightweight FHLE-RTDETR model based on real-time detection transformer (RT-DETR) to improve multi-scale disease detection and reduce model complexity in orchard environments with complex backgrounds, achieving a mean average precision (mAP@0.5) of 92.1% (Ji et al., 2025); however, disease severity evaluation was not addressed.

In summary, although significant progress has been made in peach disease detection, existing models still struggle to handle dense occlusion, large scale variation, and complex backgrounds under natural conditions. Moreover, detection models intended to support precision, target-oriented pesticide application must also incorporate reliable disease severity grading.

In this study, we focus on the detection and grading of peach leaf shot hole disease under natural conditions. A lightweight YOLO-based detection architecture is designed to achieve high-precision, real-time detection despite significant variation in disease characteristics, environmental interference, and dense leaf occlusion. In addition, a grading method for assessing the regional severity of peach leaf shot hole disease is proposed based on the detection results.

## 2 Materials

### 2.1 Data acquisition

The peach tree disease image data used in this study were collected on August 11, 2025, at the Xiaotangshan National Precision Agriculture Research and Demonstration Base in Beijing, China. Beijing has a warm temperate semi-humid monsoon climate, with high temperatures and abundant rainfall in summer; such conditions are conducive to the occurrence of peach leaf shot hole disease. The study focused on three peach varieties: *Maotao* (hairy peach), *Pantao* (flat peach), and *Youtao* (nectarine).

**Figure 1:**
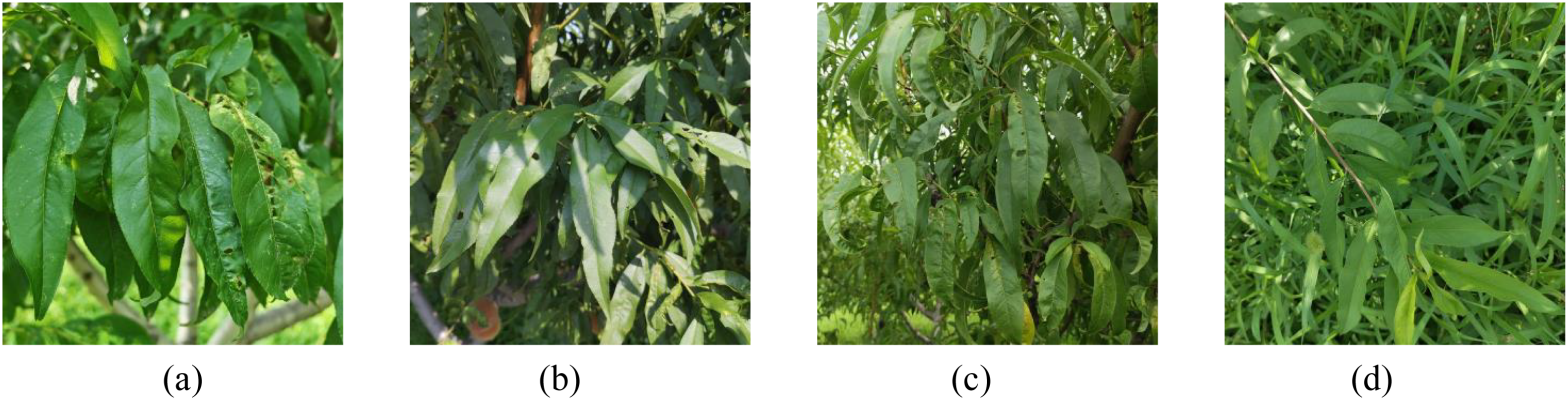
Partial images of the PSHData (peach shot hole data) dataset. **(a)** Large disease scale variation scenarios. **(b)** Inhomogeneous natural illumination scenarios. **(c)** Dense overlapping leaf scenarios. **(d)** Similar background interface scenarios.

Image acquisition was performed using multiple smartphones, including models V2301A and Magic6 Pro from the IQOO and Huawei Honor brands, respectively; the image resolutions were 3072 × 4096 pixels and 3060 × 4080 pixels, respectively. To balance image detail and global contextual information while ensuring high data quality, the distance between the imaging device and the target was maintained within a range of 20–100 cm. During data collection, disease images were captured from multiple angles at different times of day and under varying lighting conditions using handheld devices. This strategy was adopted to enhance the generalization capability of the proposed model for peach tree disease detection. A total of 837 images were collected, as shown in **Figure 1**. The dataset encompasses a wide range of real-world conditions, including large variations in disease scale, uneven natural illumination, dense leaf occlusion, and background interference from visually similar objects, thereby providing sufficient data support for subsequent model training and performance evaluation.

### 2.2 Data processing and analysis

The lightweight image annotation tool X-AnyLabeling was used to annotate the collected peach tree disease images in YOLO format. The annotated dataset was randomly divided into training, validation, and test sets at an 8:1:1 ratio, with the random seed set to 42 in Python. This study specifically targets shot hole disease on peach leaves. Among the 837 images collected at the Xiaotangshan National Precision Agriculture Research Base, 800 images exhibiting clear shot hole disease symptoms were selected and cropped to a resolution of 640 × 640 pixels.

To ensure the rigor and consistency of data annotation, multiple annotators independently annotate the samples, and the Intersection over Union between their annotations is computed to verify agreement. The annotators follow the criteria below: for leaves affected by shot-hole disease, the bounding box must enclose the entire leaf; for occluded leaves, only the visible regions are annotated; distant or blurred leaves are excluded from annotation. Discrepant cases are subsequently adjudicated and corrected by a third senior expert, thereby ensuring reliable annotation quality across the entire dataset.

**Figure 2:**
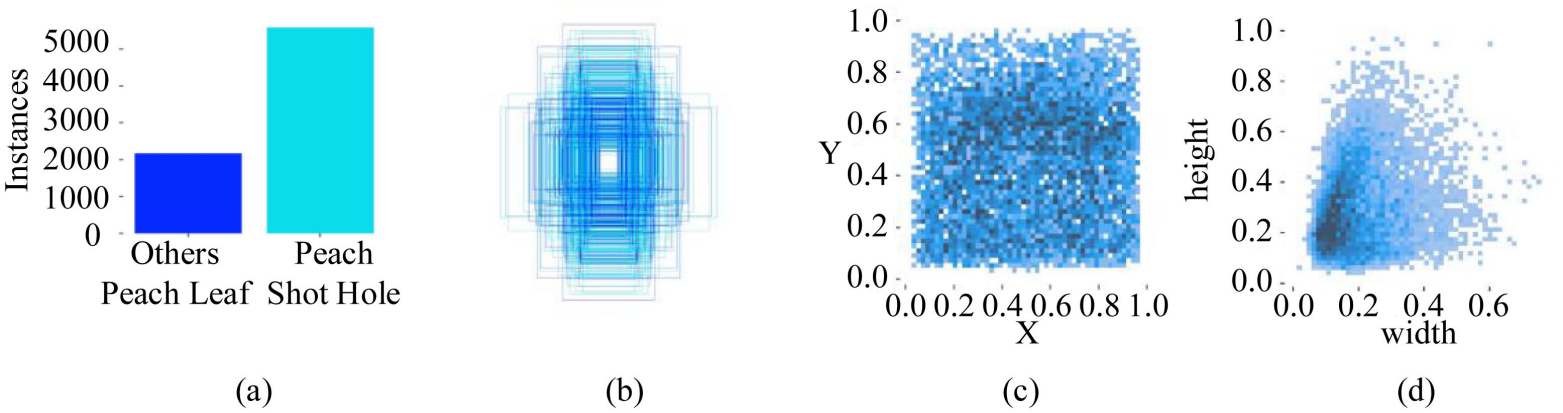
Label quantity distribution of the training set for peach leaf shot hole disease data. **(a)** The number of instances per category. **(b)** Detection box superimposition results using image center as origin. **(c)** Position of detection box center points relative to the image. **(d)** Detection box aspect ratios relative to the image.

The categories and quantities of the selected images are listed in **Table 1**, which presents the distribution of the peach leaf shot hole disease dataset. Visual data analysis techniques were applied to statistically analyze the training set. **Figure 2a** shows the number of instances for each category in the training set. **Figure 2b** shows the result of aligning and superimposing all category bounding boxes with the image center as the origin, enabling intuitive observation of the shape, size, and approximate spatial distribution of the bounding boxes. **Figure 2c** shows the distribution of target center points across the entire image, and **Figure 2d** shows the aspect ratio of targets relative to the image dimensions.

**Table 1:** Distribution of the peach leaf shot hole disease dataset.

Dataset	Other leaves	Diseased leaves (shot hole)	Proportion of other leaves	Proportion of diseased leaves
Training set	2161	5570	27.95%	72.05%
Validation set	313	541	36.65%	63.35%
Test set	250	348	41.81%	58.19%

As shown in **Figure 2**, the training set contains a total of 7731 annotated targets, including 2161 instances of other leaves and 5570 instances of peach leaf shot hole disease. The results indicate that most annotated targets are concentrated in the central region of the images. This distribution highlights the significant variability in target scales within the dataset, as well as the challenges posed by the dense distribution of small targets.

## 3 Methods

### 3.1 Construction of the peach tree disease detection model

**Figure 3:**
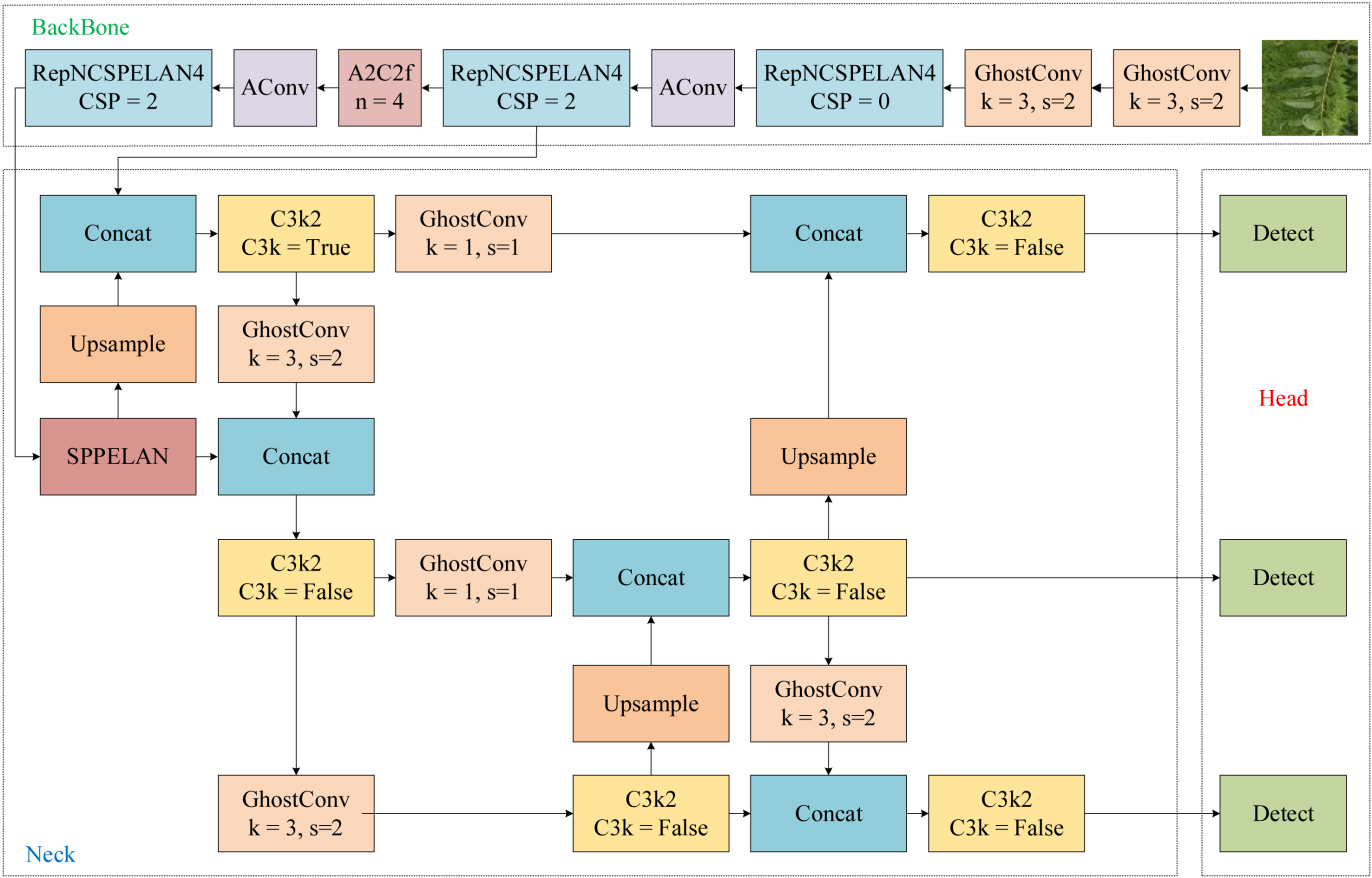
Network structure of the YOLO–PSH model.

YOLO-based models have demonstrated strong performance in real-time object detection (Redmon et al., 2016; Redmon and Farhadi, 2017; Redmon and Farhadi, 2018; Bochkovskiy et al., 2020); however, they continue to face challenges in complex peach orchard environments. Factors such as the diversity of peach tree disease types, large variations in disease scale and morphology, and complex feature distributions often result in false detections and missed detections when using conventional YOLO architectures. To address these challenges, this study proposes a novel detection framework, termed YOLO–PSH (You Only Look Once–peach shot hole), as shown in **Figure 3**.

In the backbone feature extraction network, the GhostConv and AConv modules are incorporated to reduce computational redundancy while enhancing feature compression and preserving fine-grained details. In addition, the A2C2f and RepNCSPELAN4 modules are used in combination with a regional attention mechanism and a parameter reorganization strategy to strengthen the semantic representation of critical regions and suppress background interference. In the neck component for enhanced feature aggregation, a multi-scale feature fusion structure that integrates SPPELAN (spatial pyramid pooling with efficient layer aggregation network) and C3k2 is introduced. This design improves the model’s ability to capture and adapt to overlapping and occluded features across different scales, thereby enhancing recognition performance for peach tree diseases under complex natural conditions.

### 3.2 Backbone network structure

**Figure 4:**
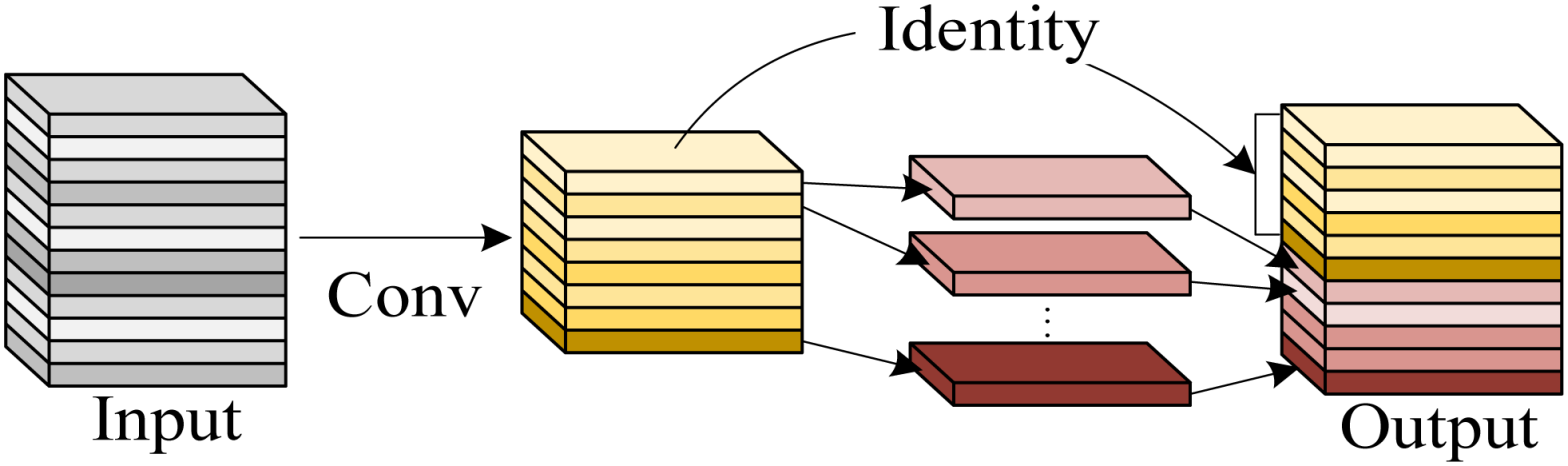
Structure of the GhostConv module.

GhostConv was proposed by Huawei Noah’s Ark Laboratory in 2020 to enable lightweight network design by utilizing feature redundancy and low-cost operations (Han et al., 2020), as shown in **Figure 4**. Its core idea is to generate ghost feature maps using inexpensive linear transformations to replace part of the standard convolution operations. This approach significantly reduces the number of parameters and computational complexity while maintaining comparable model performance.

For example, the computational complexity of a conventional convolution is given by Equation 1:

(1) $$FLOPs_{Conv}=n\cdot c\cdot k^{2}\cdot h\cdot w$$

where *n* denotes the number of output channels, *c* is the number of input channels, *k* is the convolution kernel size, and *h* × *w* is the spatial resolution of the feature map.

The computational complexity of GhostConv is expressed as Equation 2:

(2) $$FLOPs_{GhostConv}=\left(\frac{n}{s}\cdot c\cdot k^{2}+\frac{n}{s}\cdot d^{2}\cdot \left(s-1\right)\right)\cdot h\cdot w,$$

where *d* represents the kernel size of the inexpensive operation. When *s* = 2, the computational complexity can be reduced by approximately 50%.

The GhostConv module consists of the following key steps:

1. Primary feature generation: A small number of standard convolutions are applied to generate *m* intrinsic feature maps, where $m=\frac{n}{s}$, *n* is the target number of output channels, and *s* is the expansion factor.
2. Ghost feature generation: Depthwise convolution or other low-cost operations are applied to each intrinsic feature map to produce *s* − 1 ghost feature maps. These operations have significantly lower computational cost than conventional convolutions while preserving the core semantic information through linear transformations.
3. Feature concatenation: The intrinsic and ghost feature maps are concatenated along the channel dimension, resulting in *n* = *m* × *s* output feature maps and achieving the effect of “generating more from less.”

Another lightweight component of the backbone feature extraction network is the AConv module. This module adopts a progressive downsampling strategy; gentle average pooling is first applied for preliminary downsampling, followed by convolution-based downsampling. This design reduces information loss and preserves more spatial details. From a computational perspective, average pooling incurs minimal cost, and its combination with convolution enables efficient and information-preserving downsampling.

#### 3.2.1 RepNCSPELAN4 module

**Figure 5:**
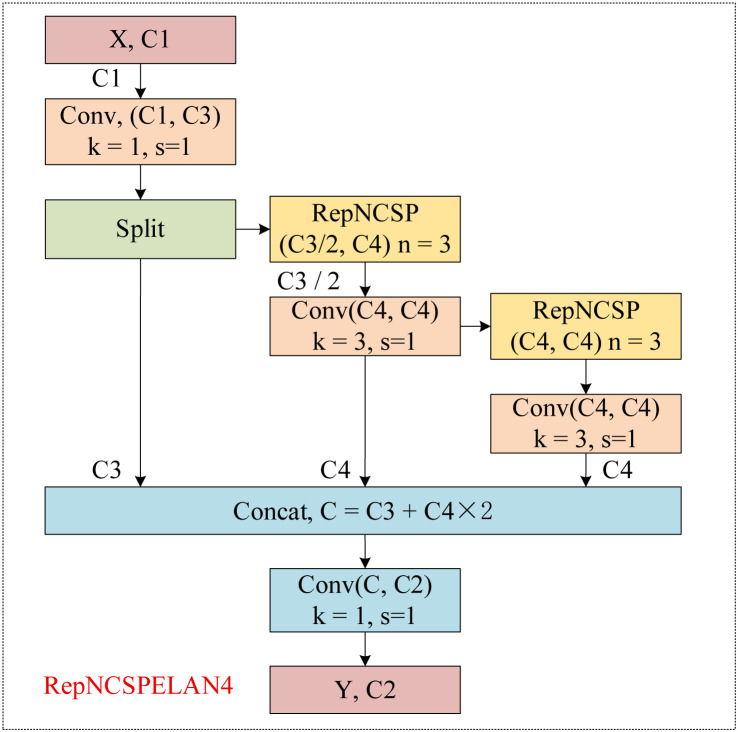
Structure of the RepNCSPELAN4 module.

RepNCSPELAN4 is a core module of the backbone network in the YOLOv9 framework (Wang et al., 2025). Based on the generalized efficient layer aggregation network, it integrates the gradient path planning of CSPNet with the feature aggregation concept of efficient layer aggregation network (ELAN), as shown in **Figure 5**. In the module name, “Rep” denotes reparameterization, while “NCSPELAN” refers to non-symmetric convolution and spatial pyramid expansion. Through multi-branch feature fusion and reparameterization, RepNCSPELAN4 achieves a balance between detection accuracy and inference speed.

The RepNCSPELAN4 module comprises the following components:

1. Input processing: Conv1 is a 1 × 1 convolution layer that compresses the input channels from *c*_1_ to *c*_3_, followed by the SiLU activation function.
2. Feature aggregation branches: Conv2 consists of RepNCSP blocks and 3 × 3 convolutions. The RepNCSP block includes multiple RepNBottleneck (reparameterized residual) blocks, which split features using a CSP structure to enhance gradient propagation efficiency. Conv3 shares the same structure as Conv2 but takes the output of Conv2 as its input, further deepening feature extraction.
3. Output fusion: Conv4 is a 1 × 1 convolution layer that concatenates the outputs of the three branches (original input, Conv2, and Conv3) and compresses them to *c*_2_ channels to generate the final feature map.

The mathematical formulation of RepNCSPELAN4 is described as follows. During training, RepConv uses three parallel branches, including a 3 × 3 convolution, a 1 × 1 convolution, and an identity mapping. The output is computed as in Equation 3:

(3) $$y=Conv_{3\times 3}\left(x\right)+Conv_{1\times 1}\left(x\right)+x.$$

During inference, these branches are merged into a single 3 × 3 convolution through an equivalent transformation. The output is computed as in Equation 4:

(4) $$W_{merged}=W_{3\times 3}+W_{1\times 1}+I,$$

where *I* denotes the identity matrix, and *W_merged_* is the reparameterized convolution kernel. The RepNBottleneck residual connection is then expressed as Equation 5:

(5) $$y=x+Conv_{3\times 3}\cdot \left(\text{SiLU}\left(Conv_{1\times 1}\left(x\right)\right)\right).$$

If the input and output channel dimensions differ, a 1 × 1 convolution is applied to the residual branch for dimension matching. Finally, the outputs of the three branches are concatenated and compressed using a 1 × 1 convolution as Equation 6:

(6) $$y=Conv_{1\times 1}\cdot \left(\text{Concat}\left(x,C_{2}\left(x\right),C_{3}\left(x\right)\right)\right).$$

#### 3.2.2 A2C2f module

**Figure 6:**
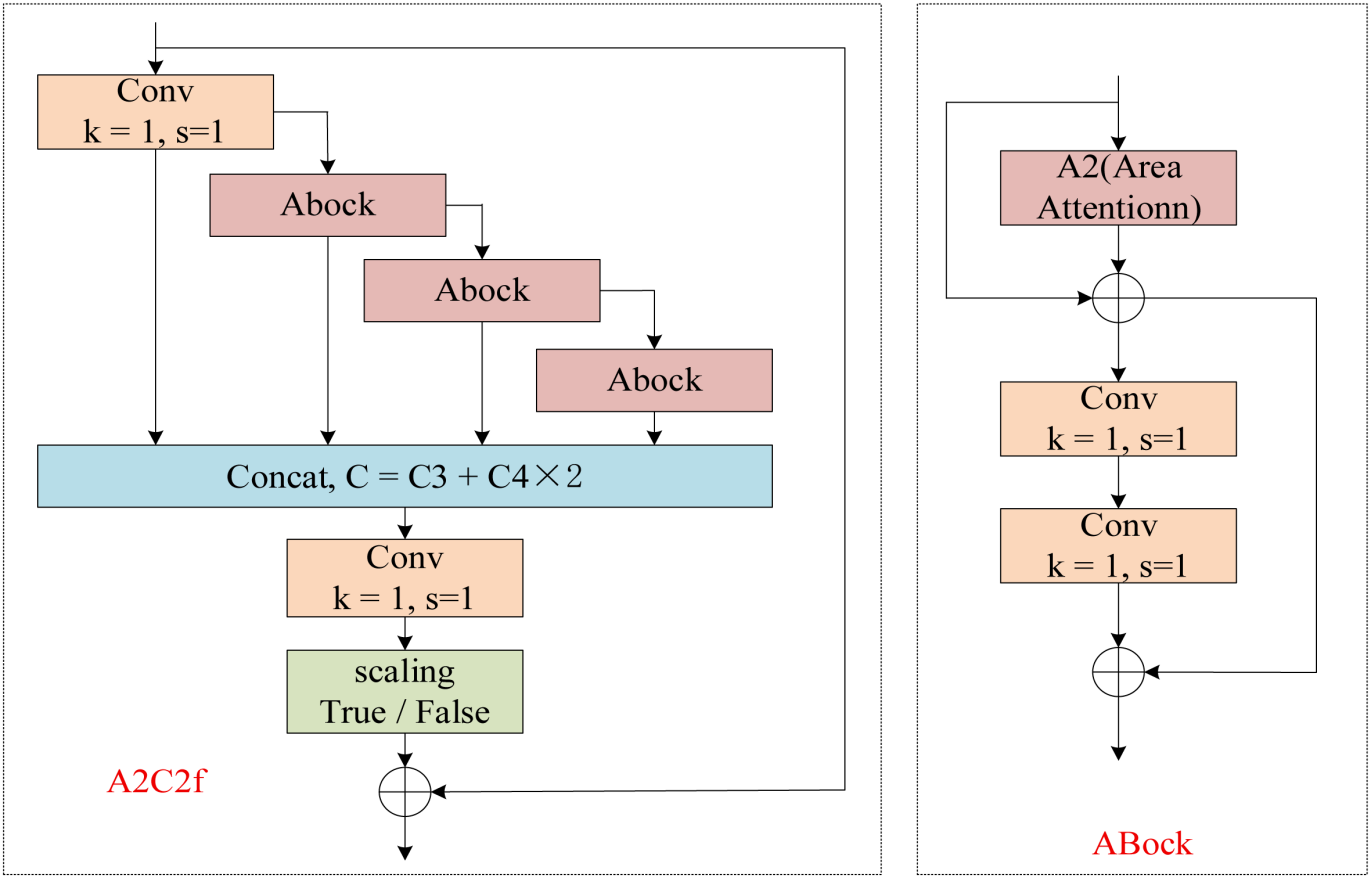
Structure of the A2C2f module.

A2C2f is an attention-enhanced feature fusion module proposed in the YOLOv12 framework (Tian et al., 2025), as shown in **Figure 6**. It aims to combine the advantages of global contextual modeling and local feature extraction while maintaining real-time inference performance. In its name, “A” denotes attention, “C” represents convolution, and “2f” refers to a two-layer feature fusion structure. The regional attention mechanism (A2) divides the feature map into simple horizontal or vertical regions, significantly reducing attention computation while preserving a large receptive field. Through regional attention and residual connections, the module enhances robustness in small-target detection and complex background scenarios.

The A2C2f module consists of the following components:

1. Input branches: cv1 is a 1 × 1 convolution layer that reduces the number of channels from 256 to 128, followed by the SiLU activation function, to decrease computational cost. cv2 is a 3 × 3 convolution layer with 256 channels and SiLU activation, used to extract richer local features.
2. Attention module (ABlock): A cross-shaped window is used to partition the feature map, and attention is computed separately in the horizontal and vertical directions to reduce global complexity.
3. Positional encoding (PE): Positional embeddings are generated via a 1 × 1 convolution and added to the attention output to enhance spatial information modeling.
4. Multi-layer perceptron (MLP): The MLP consists of two convolution layers: the first expands the channel dimension to 256, and the second compresses it back to 128, enabling feature transformation and nonlinearity.
5. Residual connection: The output of the attention module is added to the original input to mitigate gradient vanishing and promote feature reuse.

The regional attention mechanism is formulated as follows. Given an input feature map $X\in R^{H\times W\times C}$, where *H*, *W*, and *C* denote height, width, and channel number, respectively, the feature map is divided into horizontal and vertical stripes, and attention is computed independently. The output is computed as in Equation 7:

(7) $$Q_{h}=\text{Conv}\left(X,W_{q}^{h}\right),K_{h}=\text{Conv}\left(X,W_{k}^{h}\right),V_{h}=\text{Conv}\left(X,W_{v}^{h}\right).$$

The horizontal attention output is given by Equation 8:

(8) $${\text{Attn}}_{h}=\text{Softmax}\left(\frac{Q_{h}K_{h}^{T}}{\sqrt{C}}\right)V_{h}.$$

The same process is applied in the vertical direction, and the final attention output is obtained by concatenation. The output is computed as in Equation 9:

(9) $$\text{Attn}=\text{Concat}\left({\text{Attn}}_{h},{\text{Attn}}_{v}\right).$$

With residual connection, the output feature map is expressed as Equation 10:

(10) $$X_{out}=\text{MLP}\left(\text{Attn}+\text{PE}\right)+X_{in},$$

where PE denotes the positional encoding generated via a 1 × 1 convolution.

Overall, the A2C2f module captures long-range dependencies through regional attention while preserving local convolutional details. By using cross-shaped windows and linear-complexity attention, it reduces computational complexity by more than 90% compared with conventional self-attention, while improving detection accuracy for small targets and under complex background conditions.

### 3.3 Neck fusion structure

**Figure 7:**
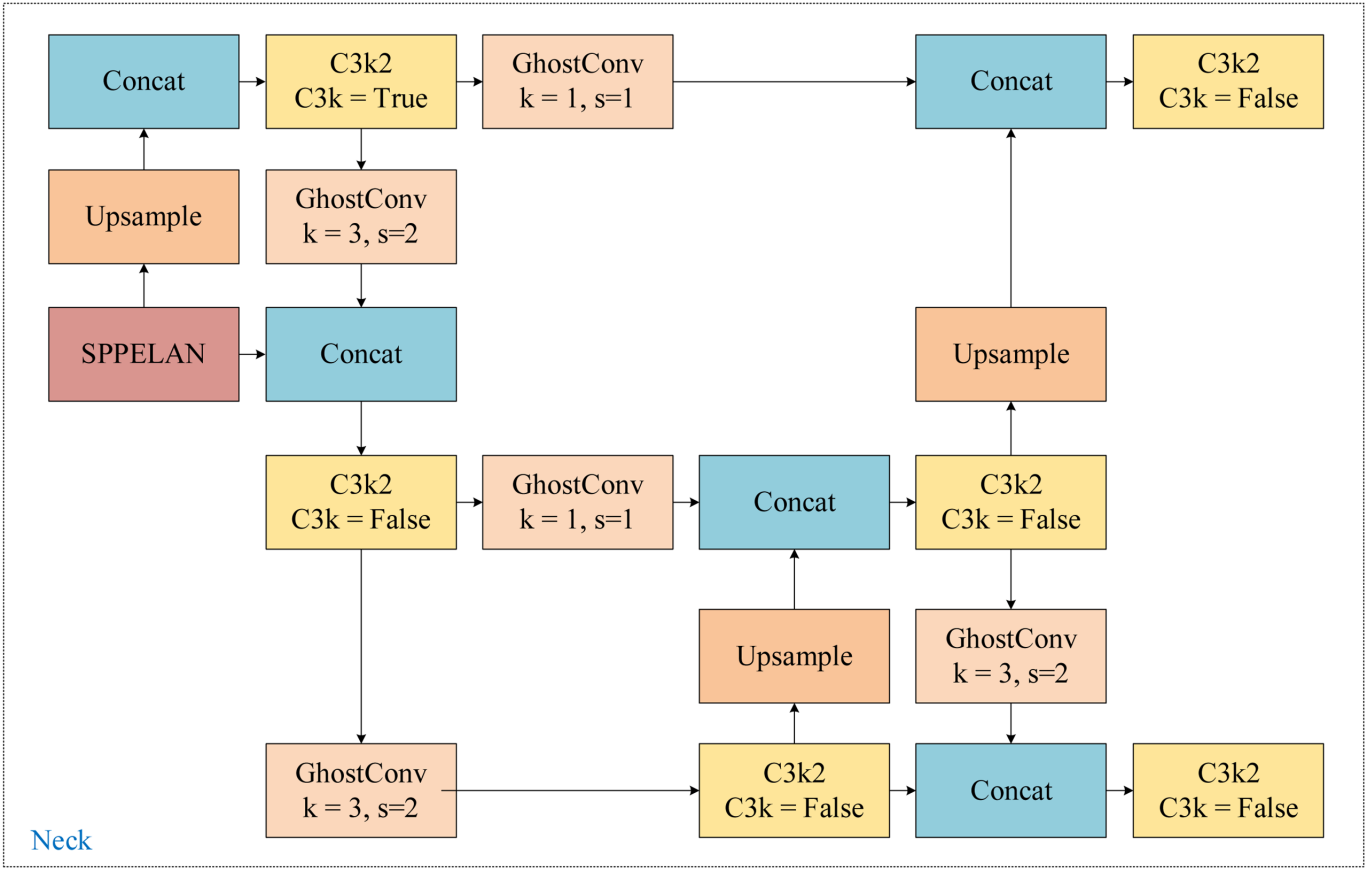
Neck feature fusion structure.

The neck component of the YOLO–PSH model embodies a fundamental innovation of this network: a shallow spatial preservation mechanism in its topological design, which distinguishes it from mainstream YOLO neck architectures. Specifically, current YOLO series and their variants generally adopt a three-layer feature fusion paradigm in the neck, extracting feature layers at scales corresponding to 80, 40, and 20 after deep downsampling from the backbone. While this design offers significant advantages in generic object detection, it presents an inherent drawback for the specialized task of peach leaf shot hole disease: the lesions are extremely small and share similar features with background noise. On the 20-scale feature map, which encodes high-level semantics, the spatial details of lesions are often indistinguishable from background noise. In contrast, the neck of YOLO–PSH takes only two shallow high-resolution feature maps—at 160 and 80 scales—from the backbone, before they undergo deep compression, as its inputs, as shown in **Figure 7**.

The theoretical basis for this modification is that shallow features preserve high-fidelity spatial details; the 160-scale feature map possesses extremely high resolution and retains the most primitive color, texture, and edge information. This enables the model to mechanistically distinguish minute perforations from background noise or stains, a capability that deep semantic features struggle to achieve.

Furthermore, the neck reconstructs the scale generation pathway: we abandon the generation using 20-scale features and instead derive a 40-scale feature layer by downsampling the 160-scale features within the neck, which is specifically dedicated to locating larger-scale intact leaves. Building on this architecture, the SPPELAN module is introduced to strengthen contextual feature representation and to fuse shallow detail features with deep semantic features through cross-scale concatenation. Lightweight GhostConv operators are incorporated to reduce computational overhead, while the C3k2 module is used to dynamically adjust channel dimensions.

The key advantages of this neck design are as follows: (1) the shallow spatial preservation mechanism with multi-scale feature fusion enables adaptation to targets of varying sizes, especially minute lesions; (2) GhostConv significantly reduces parameter count and inference latency, facilitating edge-device deployment; (3) SPPELAN enhances feature discriminability through expanded receptive fields; and (4) C3k2 balances feature complexity and computational cost. Together, these components achieve an effective trade-off between detection performance and operational efficiency.

#### 3.3.1 SPPELAN Module

**Figure 8:**
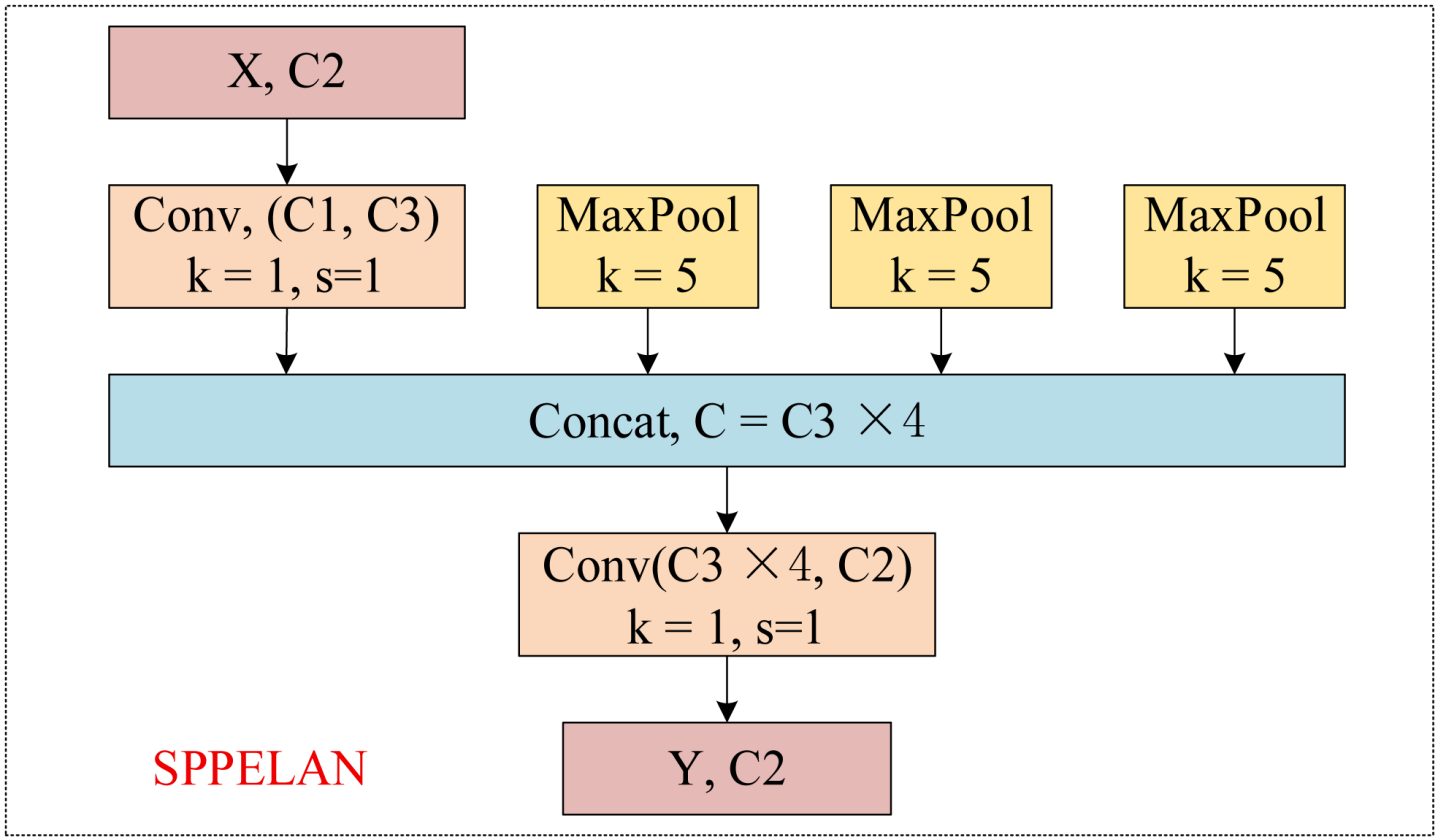
Structure of the SPPELAN module.

SPPELAN is an improved variant of the conventional spatial pyramid pooling (SPP) module (Wang et al., 2023), as shown in **Figure 8**. It integrates the strengths of SPP and ELAN. The primary objective of SPPELAN is to enhance multi-scale feature perception while maintaining computational efficiency, particularly for small targets and complex background scenarios.

The SPPELAN module consists of the following components:

1. Continuous multi-scale pooling: The input feature map is first channel-compressed via a 1 × 1 convolution (e.g., from *c*_1_ to *c*_3_), followed by three sequential 5 × 5 max-pooling operations with a stride of 1 and padding of 2, ensuring that the spatial resolution of the feature map remains unchanged.
2. Multi-scale contextual extraction: The pooling operations generate hierarchical contextual information, capturing spatial features at different granularities.
3. Feature fusion and compression: The output of the initial 1 × 1 convolution is concatenated with the three pooled feature maps along the channel dimension and then compressed to the target channel size *c*_2_ using a 1 × 1 convolution, enabling efficient feature aggregation.

The mathematical formulation of SPPELAN is as follows. Given an input feature map $X\in R^{H\times W\times C}$, the intermediate feature after channel compression is $Y={\text{Conv}}_{1\times 1}\left(X\right)$. The three sequential pooling operations are defined as Equation 11:

(11) $$Y_{1}={\text{MaxPool}}_{5\times 5}\left(Y\right),Y_{2}={\text{MaxPool}}_{5\times 5}\left(Y_{1}\right),Y_{3}={\text{MaxPool}}_{5\times 5}\left(Y_{2}\right).$$

The concatenated feature map is Equation 12:

(12) $$Y_{\text{concat}}=\text{Concat}\left(Y,Y_{1},Y_{2},Y_{3}\right)\in R^{H\times W\times 4C}.$$

After channel compression and activation, the final output is obtained as Equation 13:

(13) $$Y_{out}={\text{Conv}}_{1\times 1}\left(Y_{\text{concat}}\right)\cdot \text{SiLU}.$$

By combining multi-scale pooling with ELAN-style feature aggregation, SPPELAN effectively enhances the recall of small targets.

#### 3.3.2 C3k2 module

**Figure 9:**
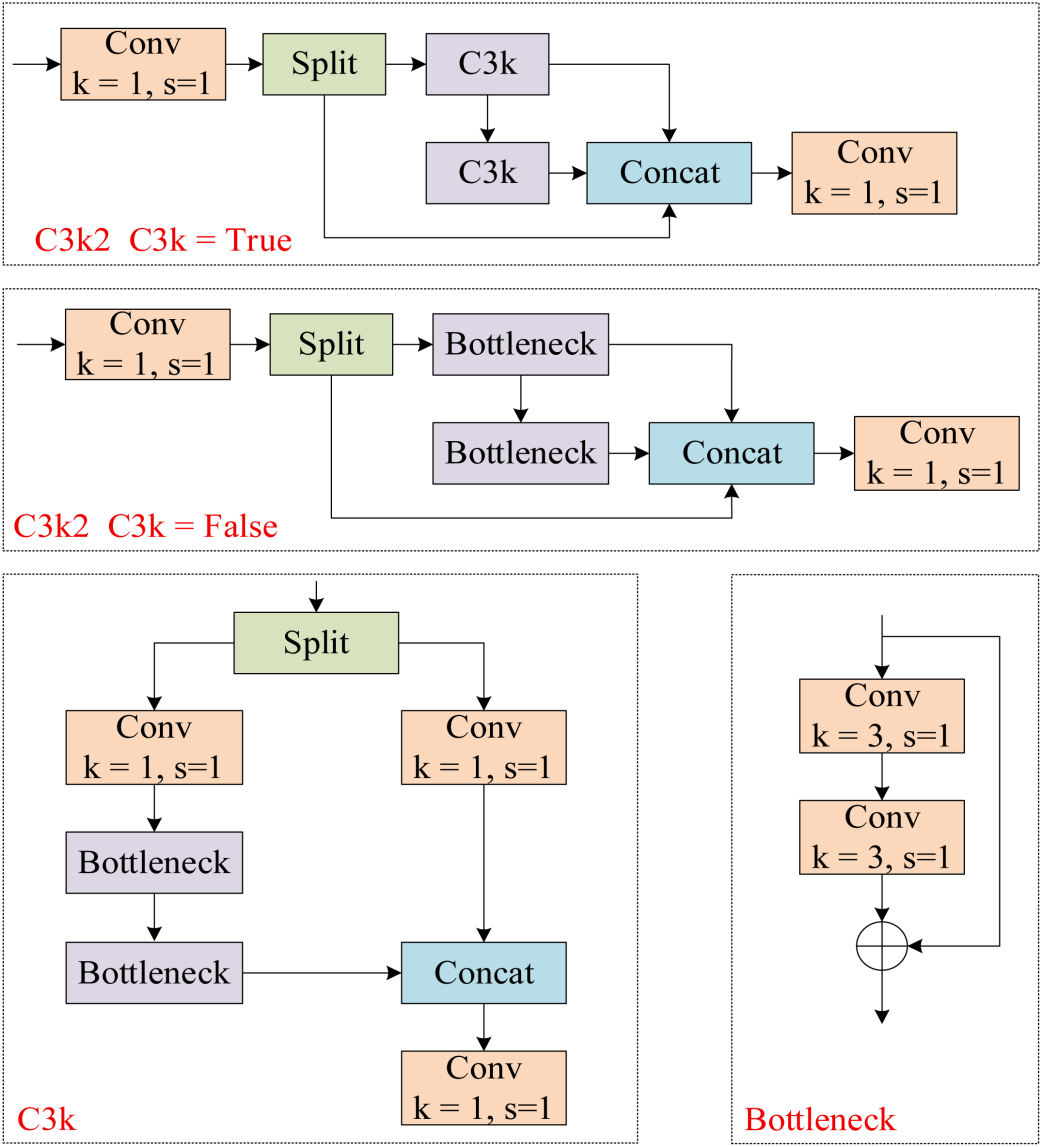
Structure of the C3k2 module.

C3k2 is an efficient feature extraction module proposed in YOLOv11 (Khanam and Hussain, 2024), designed based on C3k and parallel convolutional structures, as shown in **Figure 9**. The designation “k2” indicates the use of two convolution kernels with different sizes to enable multi-scale feature extraction.

The C3k2 module comprises the following components:

1. Dual-branch parallel processing: This includes

1.1 a direct transmission branch, in which the input feature map is channel-compressed from *c*_1_ to *c*_2_/2 using a 1 × 1 convolution, preserving shallow feature information and
1.2 a deep processing branch, in which the input feature map undergoes parallel convolution operations with kernel sizes of 3 × 3 and 5 × 5. Each branch is followed by batch normalization and the SiLU activation function to extract deeper semantic features.
Feature fusion and dynamic adjustment: The outputs of the direct transmission branch and the deep processing branch are concatenated along the channel dimension. A subsequent 1×1 convolution adjusts the number of channels to *c*_2_, and learnable weights are introduced to dynamically balance the contributions of multi-scale features.

The mathematical formulation of C3k2 is given as follows. For an input feature map $X\in R^{H\times W\times C}$, the output of the direct transmission branch is Equation 14:

(14) $$Y_{1}={\text{Conv}}_{1\times 1}\left(X\right).$$

The output of the deep processing branch is Equation 15:

(15) $$Y_{2}=\sum_{k\in \left\{3,5\right\}}{\text{Conv}}_{k\times k}\left(X\right)\cdot \text{SiLU}.$$

After feature fusion and activation, the output is Equations 16, 17:

(16) $$Y_{\text{concat}}=\text{Concat}\left(Y_{1},Y_{2}\right)\in R^{H\times W\times \left(C/2+C\right)},$$

(17) $$Y_{\text{out}}={\text{Conv}}_{1\times 1}\left(Y_{\text{concat}}\right)\cdot \text{SiLU}.$$

The C3k2 module also incorporates a lightweight design by optimizing computational complexity through group convolution (parameter *g*) and channel expansion rate (parameter *e*). For example, when *e* = 0.25, the number of hidden channels is reduced to *c*_2_ × 0.25, substantially decreasing the parameter count.

The YOLO–PSH network operates through three hierarchical stages: lightweight feature extraction, multi-scale feature aggregation, and attention-enhanced feature refinement. The model accepts an input image of size 640 × 640 × 3. In the backbone, hierarchical multi-scale features are progressively extracted by stacking GhostConv, RepNCSPELAN4, AConv, and A2C2f modules, producing feature maps with varying spatial resolutions and channel dimensions (e.g., 320 × 320 × 64 and 160 × 160 × 128). Attention mechanisms are applied to emphasize key feature dimensions, enabling preliminary semantic and detail-aware representations.

The neck constructs a bidirectional feature aggregation path based on SPPELAN and cross-scale fusion. Through upsampling and concatenation operations, combined with GhostConv and C3k2 modules, features from different backbone stages are fused. Deep semantic features are propagated to shallow layers via upsampling to support small-target detection, while shallow detail-rich features are transmitted to deeper layers through downsampling to enhance large-target representation. The inclusion of SPPELAN further expands the receptive field and strengthens information aggregation across scales.

Finally, the detection head performs object detection on three sets of feature maps at different scales (160 × 160 × 378, 80 × 80 × 672, 40 × 40 × 672), completing bounding box regression and category classification. The core strength of the YOLO–PSH model lies in its effective balance between lightweight design and detection performance. On the one hand, extensive use of GhostConv and lightweight C3 variants significantly reduces parameters and computational cost, enabling deployment under resource-constrained conditions. On the other hand, attention mechanisms and bidirectional multi-scale feature aggregation enhance feature representation and cross-scale fusion. The repeated stacking of RepNCSPELAN4 modules further deepens feature extraction, ultimately improving inference efficiency while maintaining high detection accuracy across targets of varying scales.

### 3.4 Disease severity grading method

The Chinese National Standard NY/T 1965.1–2010 specifies upper limits for pesticide application dosages across different crops and categorizes application levels into three grades: low dosage, regular dosage, and high dosage. Effective implementation of this standard requires integration with a precise disease assessment framework to ensure scientifically sound, rational, and accurate pesticide use. However, in practical agricultural production, pesticide application typically relies on regional spraying techniques, such as air-assisted spraying, where the treated area extends beyond individual leaves. Therefore, there is an urgent need for a detection approach capable of quantitatively evaluating disease severity at a regional scale rather than at the single-leaf level. To meet this need, a grading method was designed based on the management of peach leaf perforation disease. The characteristics of perforation disease control dictate that precision spraying is not conducted point-to-point on individual lesions; instead, it operates at a regional scale. Accordingly, this grading method adopts the pesticide dosage limits established by the Chinese National Standard and determines spraying decisions by incorporating the proportion of peach leaves affected by perforation disease in the target area—i.e., the percentage of diseased leaves—as jointly assessed by local peach growers and experts (Ministry of Agriculture of the People’s Republic of China, 2010).

To address this requirement, a regional disease severity grading method for peach leaf shot hole disease is proposed. First, the proportion of shot hole disease detection boxes relative to the total number of detected boxes in a single image is calculated. Specifically, the number of shot hole disease detection boxes, defined as the proportion of shot hole detection boxes (PSHDB), is divided by the total number of detection boxes across all categories in the image and multiplied by 100%. Disease severity is then classified based on this proportion. A PSHDB of 0% indicates no disease; a PSHDB between 0% and 35% indicates mild disease; a PSHDB between 35% and 70% indicates moderate disease; and a PSHDB greater than 70% indicates severe disease. As shown in the Equations 18 and 19

(18) $$Proportion\;of\;shot\;hole\;detection\;boxes=\left(\frac{N_{shot\;hole}}{N_{total\;detection\;boxes}}\right)\times 100\%$$

(19) $$Disease\;severity=\{\begin{array}{cc} No, & PSHDB=0\%\\ Mild, & 0\%<PSHDB\leq 35\%\\ Moderate & 35\%<PSHDB\leq 70\%\\ Severe, & PSHDB>70\% \end{array}$$

### 3.5 Experimental equipment and performance indicators

To verify the effectiveness of the proposed model, both ablation studies and comparative experiments with different models were conducted. The experimental environment was configured as follows: an Intel Core i9-12900K CPU operating at 3.70 GHz, 32 GB of RAM, an NVIDIA GeForce RTX 4070 Ti Super GPU with 16 GB of memory, and a 64-bit Windows operating system. Model training was implemented using the PyTorch deep learning framework (version 2.7.1).

Based on repeated experimental trials, the training hyperparameters were set as follows: the Adam optimizer was used with a cosine learning rate decay strategy, which is a widely recognized robust combination in the YOLO series; momentum was set to 0.937; weight decay was set to 5 × 10^-4^. The batch size was set to 16, it limited by GPU memory capacity and preliminary experiments verified that the loss decreased smoothly under this setting. The number of training epochs was set to 250 and was integrated with an early stopping strategy; the initial learning rate was 1 × 10^-2^; and the minimum learning rate was 1 × 10^-4^. A small-scale learning rate search conducted on this small-sample agricultural dataset confirmed that the chosen optimizer configuration guarantees stable convergence without any signs of overfitting. The peach leaf shot hole disease dataset was divided into training, validation, and test sets using an 8:1:1 ratio. All experiments were conducted under identical experimental conditions. In comparative experiments, early stopping was applied: if neither validation loss nor accuracy improved over 30 consecutive epochs, the model was considered to have converged and training was terminated (Wang et al., 2024).

Model performance was evaluated using average precision (AP), mean average precision at an intersection-over-union (IoU) threshold of 0.5 (mAP@0.5), number of parameters, FLOPs, inference time, and model size. For the proposed disease severity grading method, classification accuracy of severity levels was used as the evaluation metric. The number of parameters reflect the storage requirements of the model, while FLOPs and model size quantify its computational complexity. Collectively, these metrics provide a comprehensive assessment of the model’s detection performance, computational efficiency, and suitability for practical deployment.

## 4 Results

### 4.1 Experimental results and analysis of the YOLO–PSH model

**Figure 10:**
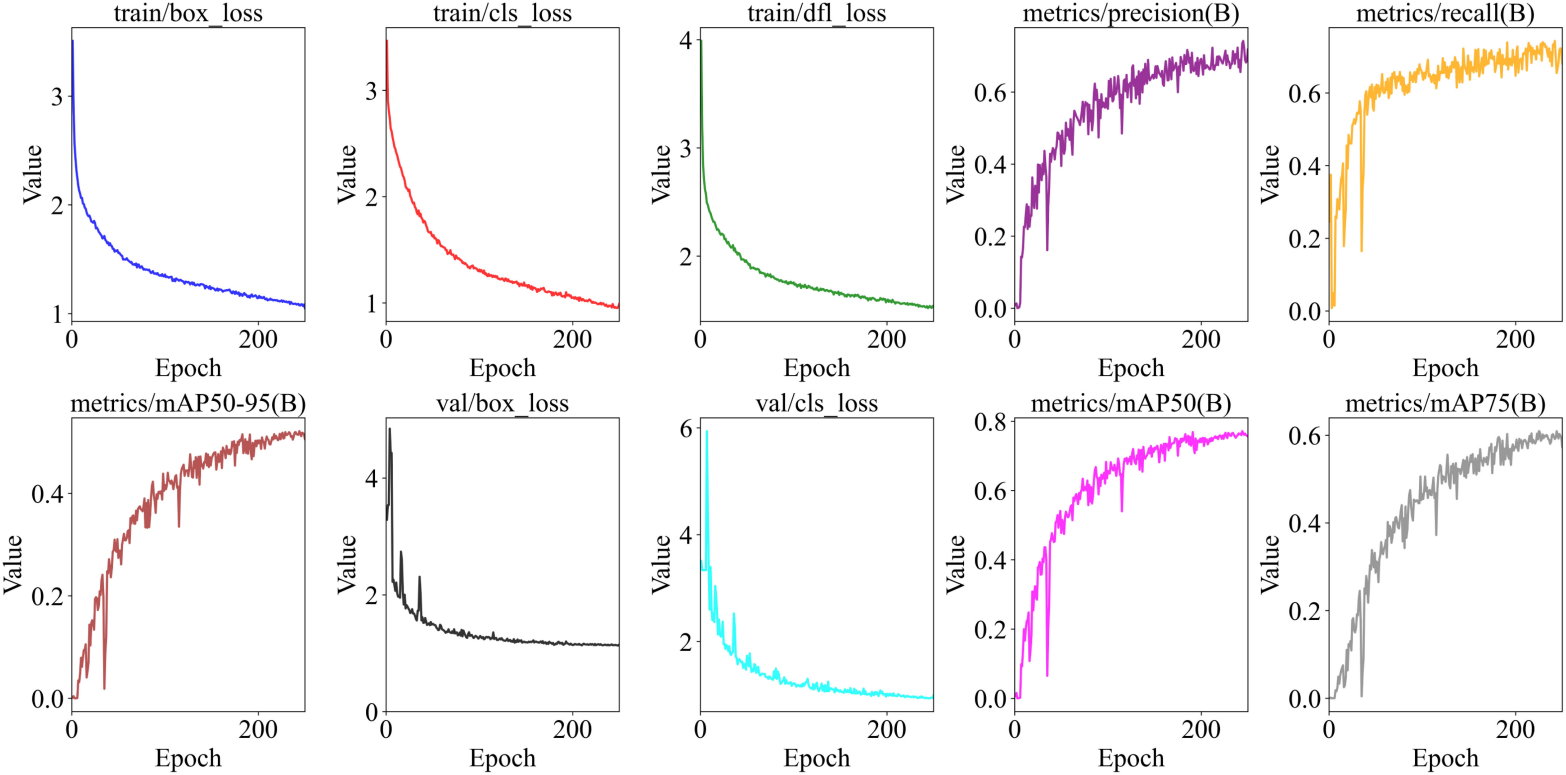
Training process and results of the YOLO–PSH model.

**Figure 10** shows the training process and performance evolution of the YOLO–PSH model. During training, the loss components (train/box_loss, train/cls_loss, and train/dfl_loss) consistently decrease and gradually stabilize, indicating steady convergence of the regression and classification objectives on the training set. With respect to validation performance, val/box_loss and val/cls_loss exhibit minor fluctuations during the early training stages but subsequently decrease and stabilize, following trends similar to those observed for the training losses. This behavior suggests that the model does not exhibit evident overfitting. Regarding detection performance metrics, precision, recall, and average precision under different IoU thresholds (including mAP@0.5–0.95, mAP@0.5, and mAP@0.75) increase steadily with the number of training epochs and converge in the later stages of training. These trends demonstrate progressive improvements in detection accuracy, recall capability, and overall detection performance for peach leaf shot hole disease, ultimately reaching a stable and reliable detection level.

### 4.2 Ablation experiment results and analysis

Ablation experiments were conducted to evaluate the effectiveness of each proposed component in the YOLO–PSH model. By systematically adding or removing individual modules and observing the resulting performance changes, the contributions of both individual components and their combinations can be quantitatively assessed. In this study, ablation experiments were designed using peach leaf shot hole disease as the detection target to verify the effectiveness of the introduced modules, including GhostConv, AConv, A2C2f, RepNCSPELAN4, SPPELAN, and the head structure. The experimental results are presented in **Tables 2**, **3**. A baseline detection model without any additional modules was used as a reference. The baseline network (None) is constructed by replacing all additionally added modules in YOLO-PSH with ordinary Conv modules. Seven groups of comparative experiments were constructed by progressively introducing the proposed modules. AP、mAP@0.5 were used as detection performance metrics, while FLOPs and Model size were adopted to evaluate computational complexity. This experimental design enables a systematic analysis of the impact of each module on both model accuracy and efficiency.

**Table 2:** Ablation experiment results.

Module	Used	Used	Used	Used	Used	Used	AP/%	mAP@0.5/%	FLOPs /GFLOPs	Model size/MB
None	×	×	×	×	×	×	74.08	67.79	48.63	12.64
GhostConv	✓	×	×	×	×	×	78.92	74.15	34.35	8.32
AConv	✓	✓	×	×	×	×	77.87	73.81	2.98	7.67
A2C2f	✓	✓	✓	×	×	×	78.37	71.89	7.82	6.46
RepNCSPELAN4	✓	✓	✓	✓	×	×	80.21	74.36	9.22	7.51
SPPELAN	✓	✓	✓	✓	✓	×	80.91	74.95	9.31	7.61
Neck model	✓	✓	✓	✓	✓	✓	81.49	76.90	8.43	7.00

**Table 3:** Individual modules experiment results.

Module	Used	Used	Used	Used	Used	Used	AP/%	mAP@0.5/%	FLOPs/GFLOPs	Model size/MB
GhostConv	✓	×	×	×	×	×	75.01	67.11	47.63	12.46
AConv	×	✓	×	×	×	×	78.60	70.49	4.37	11.30
A2C2f	×	×	✓	×	×	×	76.69	69.90	38.36	10.67
RepNCSPELAN4	×	×	×	✓	×	×	78.06	71.75	54.76	13.70
SPPELAN	×	×	×	×	✓	×	77.88	70.14	48.88	12.71
Neck model	×	×	×	×	×	✓	80.42	74.90	22.47	5.82

As shown in **Tables 2**, **3**, the progressive introduction of individual modules results in an overall trend of simultaneous performance improvement and computational complexity reduction. The contributions of each module are summarized as follows.

Effect of the GhostConv module: When only the GhostConv module was introduced (Experiment 2), detection accuracy increased from 74.08% (baseline) to 78.92%, mAP@0.5 improved from 67.79% to 74.15%, FLOPs decreased from 48.63 to 34.35, and model size decreased from 12.64 to 8.32. These results demonstrate that GhostConv significantly reduces computational complexity while improving detection accuracy, providing a strong lightweight foundation for subsequent module integration.

Complexity optimization by the AConv module: After incorporating the AConv module on top of GhostConv (Experiment 3), accuracy marginally decreased to 77.87%, while mAP@0.5 reached 73.81%. Notably, FLOPs dropped sharply to 2.98 and model size dropped sharply to 7.67. Although a minor reduction in accuracy was observed, AConv substantially enhanced computational efficiency and detection stability, confirming its effectiveness in reducing model complexity.

Performance enhancement by the A2C2f module: With the introduction of the A2C2f module (Experiment 4), accuracy recovered to 78.37%, while mAP@0.5 decreased to 71.89%. FLOPs increased to 7.82 but remained at a relatively low level. These results indicate that A2C2f enhances feature representation and lesion recognition while maintaining acceptable computational complexity.

Fine-grained optimization by RepNCSPELAN4 and SPPELAN modules: After adding the RepNCSPELAN4 module (Experiment 5), accuracy further increased to 80.21%, mAP@0.5 improved to 74.36%, and FLOPs increased to 9.22. With the subsequent introduction of the SPPELAN module (Experiment 6), mAP@0.5 increased to 74.95%, FLOPs marginally increased to 9.31, and accuracy remained at 80.91%. These modules enhance feature transmission efficiency and structural robustness with minimal impact on computational cost, providing a stable foundation for the final detection head.

Overall performance of the complete YOLO–PSH model: Experiment 7 integrates all proposed modules to form the complete YOLO–PSH model. Compared with the baseline, detection accuracy increases by 7.41 percentage points, mAP@0.5 improves by 9.11 percentage points, model size decrease by 5.6 MB and FLOPs decrease by 40.2, corresponding to an 82.64% reduction in computational complexity. These results clearly demonstrate the strong synergistic optimization achieved by the proposed architecture, enabling substantial performance gains while maintaining an extremely lightweight design. The YOLO–PSH model, therefore, satisfies the dual requirements of high detection accuracy and efficient deployment in real-world orchard environments.

### 4.3 Comparative experiment results and analysis

To verify the effectiveness of the proposed model for peach leaf shot hole disease detection, this study conducted a comparative analysis of seven mainstream vision models, including YOLOv8, YOLOv9, YOLOv10, YOLOv11, YOLOv12, YOLOv13 (Lei et al., 2025; Li et al., 2022; Wang et al., 2024), and the proposed YOLO–PSH. Model performance was evaluated across three dimensions: detection accuracy (AP and mAP@0.5), lightweight characteristics (number of parameters, FLOPs, and model size), and inference efficiency (inference time). The comparative results are presented in **Table 4**.

**Table 4:** Detection results of different models.

Model	AP/%	mAP@0.5/%	Parameters/M	FLOPs/GFLOPs	Model size/MB	Inference/ms
YOLOv8	79.51	75.02	3.15	8.86	12.08	4.5
YOLOv9	79.27	74.51	2.12	7.85	7.71	6.4
YOLOv10	75.97	70.20	2.71	8.74	10.67	3.5
YOLOv11	78.15	70.74	2.62	6.61	10.07	5.3
YOLOv12	76.65	70.02	2.55	5.98	9.70	6.6
YOLOv13	78.55	73.86	2.46	6.43	5.16	10.4
YOLO–PSH	81.49	76.90	1.82	8.71	7.00	7.1

In terms of detection accuracy, YOLO–PSH achieves the best performance, with an AP of 81.49% and an mAP@0.5 of 76.90%. At the same time, it exhibits strong lightweight characteristics, with only 1.82 million parameters and a model size of 7.00 MB. This superior performance can be attributed to its architecture, which is specifically optimized for disease detection and is therefore more effective at capturing the morphological characteristics of peach leaf shot hole disease lesions and maintaining high sensitivity under complex background conditions.

YOLOv8 ranks second, with an AP of 79.51% and an mAP@0.5 of 75.02%. Although its parameter count is higher than that of YOLO–PSH, its shortest inference time of 4.5 ms provides a clear advantage in real-time detection scenarios. Its efficient feature extraction and inference mechanisms enable a favorable balance between detection speed and accuracy. YOLOv9 ranks third, achieving an AP of 79.27% and an mAP@0.5 of 74.51%, with only 2.12 million parameters. This result demonstrates a good balance between accuracy and lightweight design, indicating strong adaptability of its general architecture to leaf disease detection tasks.

YOLOv13 achieves an mAP@0.5 of 73.86%, marginally lower than that of YOLOv9, but maintains relatively low parameter count and FLOPs, suggesting effective lightweight optimization while preserving detection accuracy. YOLOv11 and YOLOv12 exhibit similar mAP@0.5 performance, at 70.74% and 70.02%, respectively. Both models achieve low computational complexity, with FLOPs of 6.61 and 5.98, reflecting the effectiveness of simplified network structures in meeting the core feature extraction requirements of peach leaf shot hole disease detection, particularly in resource-constrained deployment scenarios.

**Figure 11:**
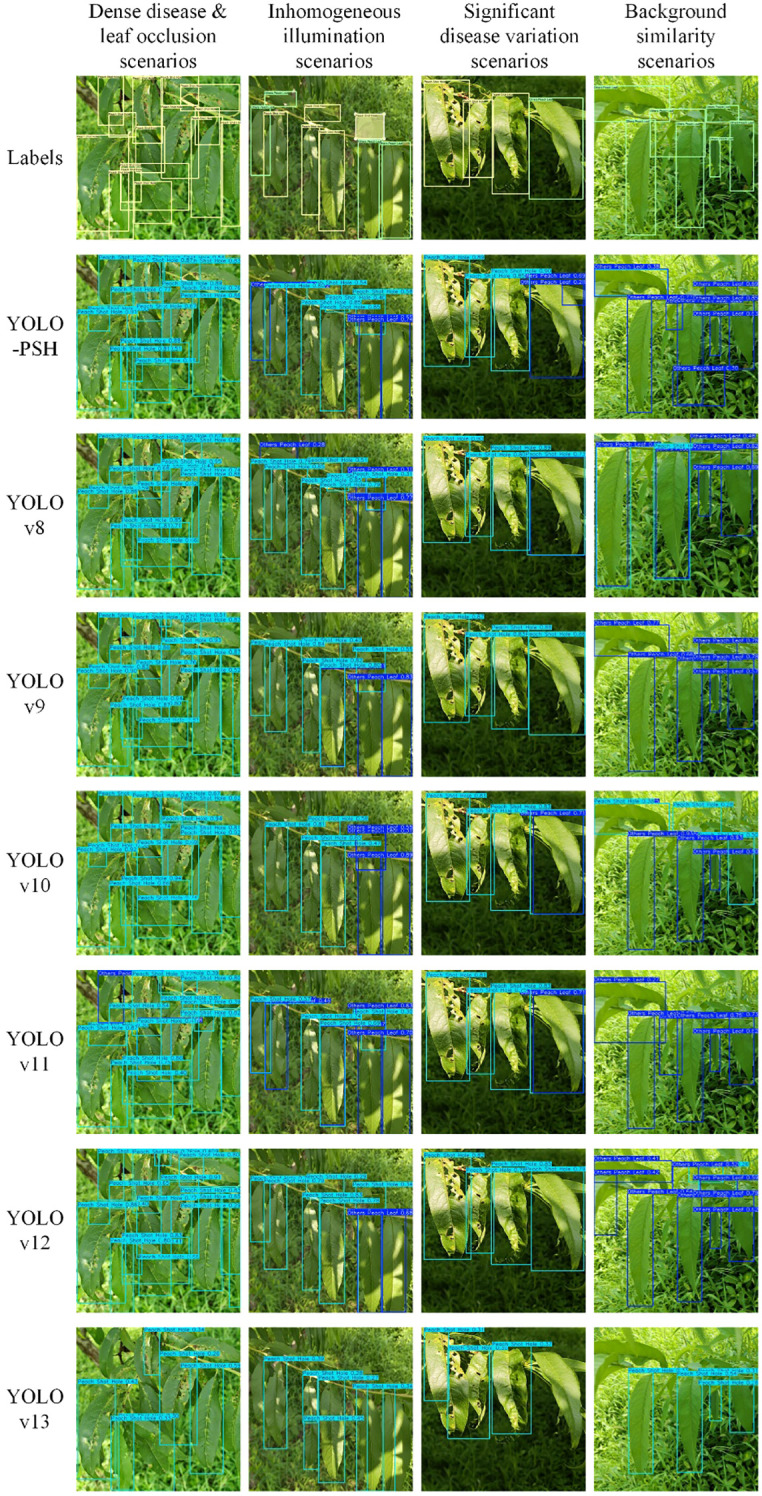
Detection results of peach leaf shot hole disease.

Overall, compared with other object detection networks, YOLO–PSH demonstrates superior performance in terms of detection accuracy and deployment suitability. To further validate its effectiveness in real-world applications, randomly selected images from the test set were used for qualitative evaluation, as shown in **Figure 11**.

As shown in **Figure 11**, YOLO–PSH exhibits clear advantages in complex environmental scenarios with large scale variation, uneven natural illumination, dense leaf occlusion, and visually similar background interference. Compared with other models, it demonstrates stronger robustness and generalization capability, effectively reducing missed detections while avoiding false positives and redundant detections.

### 4.4 Experimental results and analysis of disease severity detection

**Figure 12:**
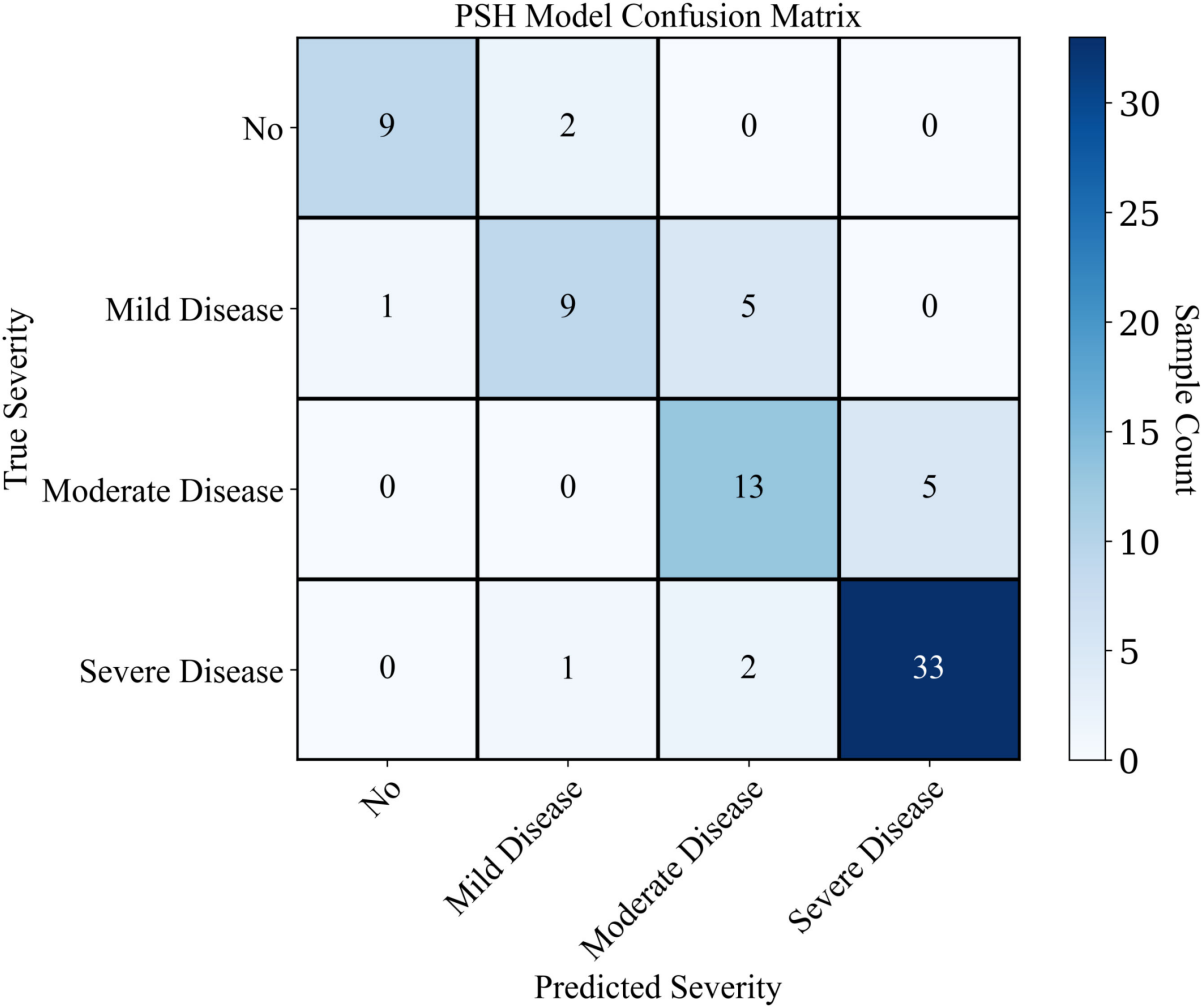
Confusion matrix of disease severity grading for YOLO–PSH.

**Figure 13:**
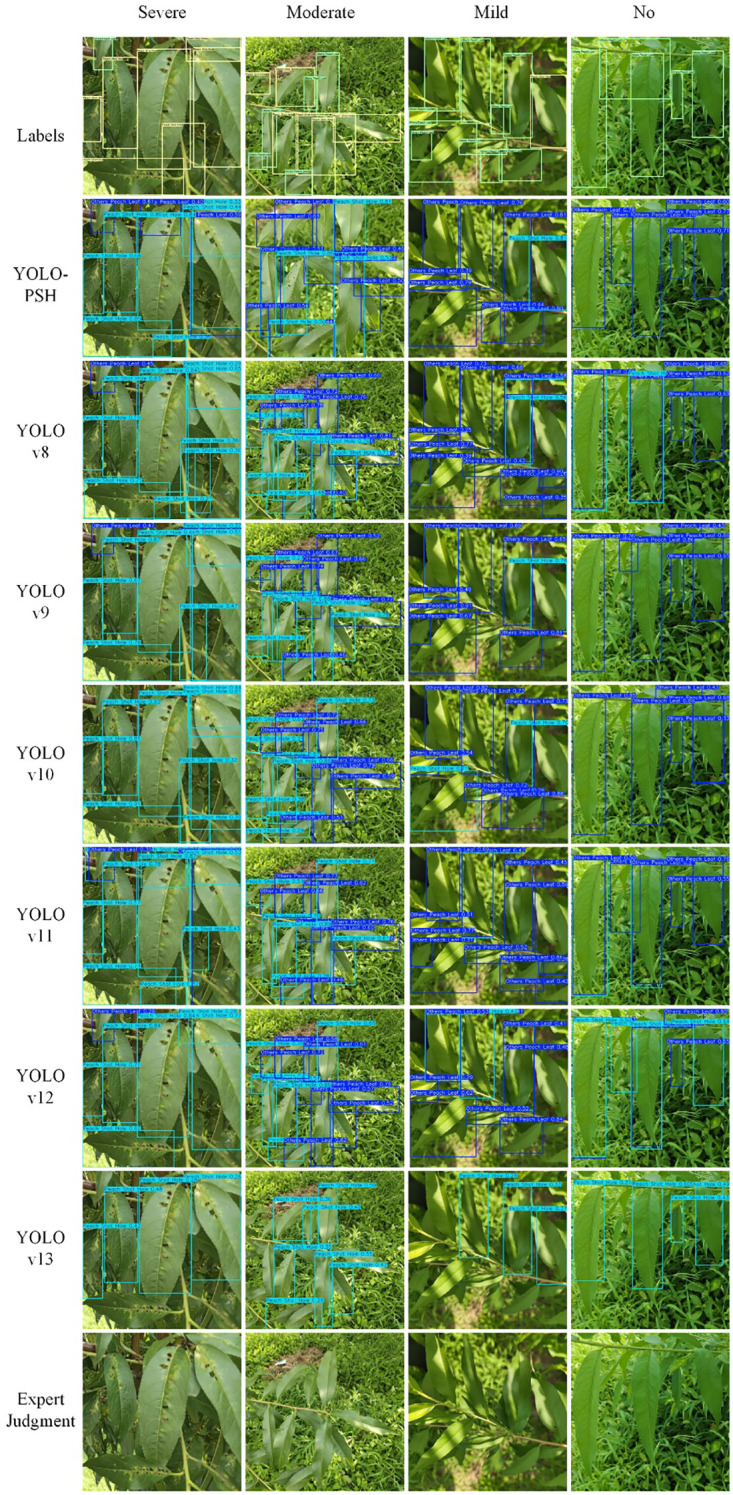
Grading results of peach leaf shot hole disease severity.

To evaluate the performance of different YOLO-series models in detecting varying severities of peach leaf shot hole disease, this experiment considered four severity levels (no disease, mild disease, moderate disease, and severe disease) and compared the detection accuracy of seven models. The experimental results are presented in **Figures 12**, **13**, with quantitative accuracy values listed in **Table 5**.

**Table 5:** Detection performance for different severities of peach leaf shot hole disease.

Model	No/%	Mild/%	Moderate/%	Severe/%	Overall/%
YOLOv8	81.82	60.00	66.67	88.89	77.50
YOLOv9	72.73	66.67	66.67	91.67	78.75
YOLOv10	72.73	40.00	72.22	88.89	73.75
YOLOv11	63.64	53.33	61.11	83.33	70.00
YOLOv12	72.73	53.33	66.67	80.56	71.25
YOLOv13	63.64	46.67	61.11	86.11	70.00
YOLO–PSH	81.82	60.00	72.22	91.67	80.00

The results indicate that YOLO–PSH achieves the most balanced and overall best performance across severity categories. It attains leading accuracy for no disease, moderate disease, and severe disease, and demonstrates a particularly strong advantage in detecting mild disease, which represents the most challenging category owing to subtle and atypical symptoms. YOLOv9 and YOLOv12 show comparable performance and outperform YOLO–PSH in detecting moderate and severe disease; however, their accuracy in detecting mild disease is relatively low. YOLOv13 exhibits unstable performance, achieving extremely high accuracy only for severe disease while failing to reliably detect the other severity categories.

Across all models, severe disease generally presents lower detection difficulty owing to its prominent visual characteristics, whereas mild disease remains the most challenging category because of weak and ambiguous lesion features. Overall, these findings demonstrate that YOLO–PSH is well-suited for field deployment and early warning of peach leaf shot hole disease. Improving detection accuracy for moderate disease severity remains a critical direction for future model optimization.

## 5 Discussion

This study focuses on the detection of peach leaf shot hole disease and proposes a novel YOLO–PSH framework. Through the coordinated design of GhostConv, RepNCSPELAN4, A2C2f, AConv, and multi-scale bottleneck fusion modules, the proposed model achieves a favorable balance between lightweight architecture and high detection accuracy. To address the challenges of peach leaf shot hole disease detection under natural conditions, such as diverse lesion morphologies, large scale variations, leaf overlap, occlusion, and complex background interference, a series of targeted design strategies was implemented. These include lightweight feature extraction module replacement, refined fusion of high- and low-frequency information, and multi-scale receptive field construction. Together, these strategies form an end-to-end optimization pipeline from input enhancement to feature refinement and output improvement, thereby enhancing detection robustness and stability.

In addition, to meet the practical requirements of precision disease prevention and control in real agricultural environments, this study proposes a regional disease severity grading method for peach leaf shot hole disease. This method provides essential technical support for differentiated disease management and precision pesticide application. The design rationale and practical value of the proposed approach are discussed as follows.

(1) Effectiveness of architectural design and module integration: Ablation experiments demonstrate that replacing standard convolution layers with GhostConv significantly reduces model parameters and computational complexity while maintaining or improving detection performance. The RepNCSPELAN4 and A2C2f modules further enhance feature discrimination by refining high- and low-frequency information and focusing on lesion details, thereby enhancing accuracy gains. The neck architecture integrates dual-branch feature fusion and bidirectional downsampling, incorporating a multi-scale fusion strategy based on SPPELAN and C3k2. These complementary module combinations enable performance improvements while preserving a lightweight design. Compared with standard YOLO architectures, YOLO–PSH exhibits superior adaptability in complex scenarios involving leaf overlap, occlusion, and background interference.

(2) Comparison with existing peach disease detection methods: Compared with the improved YOLOv5s model proposed by Yao et al (Yao et al., 2024), YOLO–PSH achieves improvements of 9.2%, 14.3%, and 11.7% in accuracy, recall, and mAP@0.5, respectively. These gains primarily result from the precise lesion-focused feature extraction enabled by RepNCSPELAN4 and A2C2f, as well as the enhanced multi-scale perception provided by the neck fusion structure.

Li investigated peach disease identification based on deep learning and achieved basic disease classification (Li, 2023); however, the model lacked optimization for multi-scale lesion characteristics and complex background interference, resulting in lower detection accuracy and recall under real field conditions than those achieved by YOLO–PSH. Wang proposed a convolutional neural network-based peach leaf disease recognition algorithm emphasizing accuracy improvement (Wang, 2023), but its relatively complex architecture did not consider real-time performance, limiting its applicability for field deployment. In contrast, YOLO–PSH achieves rapid detection responses through lightweight design while maintaining high accuracy.

Sun constructed a peach leaf disease dataset for deep learning–based image recognition (Sun, 2022), but the dataset scale and diversity were limited. By contrast, YOLO–PSH is trained on a natural-scene dataset, effectively compensating for this limitation. Its lightweight design enables deployment on low-power devices, such as mobile field inspection equipment, thereby expanding its application scope and practical value.

Compared with the improved YOLOv5su model by Yao et al (Yao et al., 2024), YOLO–PSH again demonstrates improvements of 9.2%, 14.3%, and 11.7% in accuracy, recall, and mAP@0.5, respectively. These advantages are largely attributed to the A2C2f module, which enhances lesion-focused attention and effectively suppresses interference from complex peach leaf backgrounds, such as vein textures and illumination variations. Furthermore, compared with the PCNN-IPELM method proposed by Huang et al (Huang et al., 2020), YOLO–PSH improves detection accuracy by 3.8% and exhibits superior generalization to its lightweight design and high-quality dataset, making it more suitable for deployment on resource-constrained hardware.

(3) Advantages of the proposed disease severity grading method: Conventional grading of peach leaf shot hole disease relies on manual assessment of lesion area and quantity, which is subjective, difficult to quantify, and highly dependent on individual experience. Most existing studies on peach disease detection emphasize accuracy improvement, while those involving severity grading often depend on manually annotated lesion area ratios and fail to achieve end-to-end integration of detection and grading. For example, Hu et al. proposed an improved Mask R-CNN-based method for disease region segmentation (Hu et al., 2023); however, the model is computationally complex and does not include a grading mechanism, limiting its applicability for full-process precision disease management.

The grading method proposed in this study establishes a quantitative criterion based on the percentage of shot hole disease detection boxes relative to the total number of peach leaf detection boxes. This approach directly links disease severity categories (no disease, mild, moderate, and severe) to model detection outputs, achieving an overall grading accuracy of 80%, which outperforms other YOLO-series models. By enabling automated and quantitative disease grading, this method eliminates subjective errors associated with manual assessment. Moreover, the high detection accuracy of YOLO–PSH ensures the reliability of the grading basis, providing a solid foundation for precision disease management and pesticide application.

Despite the promising performance of the proposed YOLO–PSH model, several limitations remain.

1. First, the current study focuses on a single crop (peach), and the model’s detection performance under extreme weather conditions and rare disease symptoms has not been thoroughly evaluated; future work will aim to enhance robustness and generalization in highly variable environments through multi-crop disease datasets and transfer learning. Additionally, external public datasets will be introduced for validation, elevating the single-scenario model into a universally applicable model.
2. Second, the current early disease warning capability is limited, which can be strengthened by integrating multi-source data, including meteorological, soil, and hyperspectral information.
3. Third, practical deployment in real orchard environments is an essential future step, requiring optimization for edge devices and on-site real-time inference. In addition, in-depth research will be conducted on peach leaf disease detection using the Transformer-based RT-DETR model under natural conditions.
4. Finally, fine-grained analysis of individual leaf lesions, such as lesion counting and severity assessment, is needed to deliver more precise diagnostic information, which constitutes a direction for our future research.

## 6 Conclusions

This study addresses the challenges of low detection accuracy for peach leaf shot hole disease caused by substantial morphological variability, diverse lesion types, and complex growing environments, as well as the high computational cost, slow inference speed, and limited lightweight design of conventional deep learning models in real-world settings. A YOLO–PSH detection framework is developed, together with a regional disease severity grading method for peach leaf shot hole disease under natural conditions. The proposed approach enables accurate disease detection and reliable severity assessment in complex orchard environments.

Through structural optimization of the detection network, the proposed method achieves both high detection accuracy and efficient inference. Experimental results demonstrate that the AConv module enhances disease feature compression and detail preservation by combining pooling-based dimensionality reduction with convolutional feature extraction. Integration of the GhostConv module further enables a lightweight architecture while maintaining strong performance, achieving an AP of 81.49% with efficient inference. By utilizing a self-constructed dataset and a multi-scale feature fusion strategy, the proposed model improves the representation of key disease features and exhibits consistent detection performance across lesions of varying scales. This capability effectively addresses the limitations of manual disease identification in complex natural environments and provides a reliable basis for disease severity evaluation.

Based on the YOLO–PSH detection framework, a quantitative disease severity grading method is proposed to enable precise grading of peach leaf shot hole disease under natural conditions. Experimental results show that the YOLO–PSH model achieves an mAP@0.5 of 76.90% with an inference time of only 7.1 ms per image. The proposed grading method attains an overall accuracy of 80%, outperforming existing YOLO-series models. Its grading logic is simple, quantifiable, and objective, effectively eliminating the subjectivity inherent in conventional manual grading. As a result, the method provides critical technical support for precision pesticide application and differentiated disease prevention and control strategies for peach leaf shot hole disease.

Future work will focus on expanding dataset diversity, further enhancing model robustness, and promoting the practical deployment of the proposed approach in intelligent agricultural disease detection systems.

## Data Availability

The original contributions presented in the study are included in the article/supplementary material. Further inquiries can be directed to the corresponding authors.
